# Tuberculosis Detection in Paratuberculosis Vaccinated Calves: New Alternatives against Interference

**DOI:** 10.1371/journal.pone.0169735

**Published:** 2017-01-10

**Authors:** Miriam Serrano, Natalia Elguezabal, Iker A. Sevilla, María V. Geijo, Elena Molina, Rakel Arrazuria, Alfonso Urkitza, Gareth J. Jones, Martin Vordermeier, Joseba M. Garrido, Ramón A. Juste

**Affiliations:** 1 NEIKER-Tecnalia, Basque Institute for Agricultural Research and Development, Animal Health Department, Derio, Bizkaia, Spain; 2 Bovine Practitioner, Gernika, Bizkaia, Spain; 3 TB Immunology and Vaccinology, Department of Bacteriology, APHA, Surrey, United Kingdom; 4 SERIDA, Agri-food Research and Development Regional Service, Villaviciosa, Asturias, Spain; University of Minnesota, UNITED STATES

## Abstract

Paratuberculosis vaccination in cattle has been restricted due to its possible interference with the official diagnostic methods used in tuberculosis eradication programs. To overcome this drawback, new possibilities to detect *Mycobacterium bovis* infected cattle in paratuberculosis vaccinated animals were studied under experimental conditions. Three groups of 5 calves each were included in the experiment: one paratuberculosis vaccinated group, one paratuberculosis vaccinated and *M*. *bovis* infected group and one *M*. *bovis* infected group. The performance of the IFN-gamma release assay (IGRA) and the skin test using conventional avian and bovine tuberculins (A- and B-PPD) but also other more specific antigens (ESAT-6/CFP10 and Rv3615c) was studied under official and new diagnostic criteria. Regarding the IGRA of vaccinated groups, when A- and B-PPD were used the sensitivity reached 100% at the first post-challenge sampling, dropping down to 40–80% in subsequent samplings. The sensitivity for the specific antigens was 80–100% and the specificity was also improved. After adapting the diagnostic criteria for the conventional antigens in the skin test, the ability to differentiate between *M*. *bovis* infected and non-infected animals included in paratuberculosis vaccinated groups was enhanced. Taking for positive a relative skin thickness increase of at least 100%, the single intradermal test specificity and sensitivity yielded 100%. The comparative intradermal test was equally accurate considering a B-PPD relative skin increase of at least 100% and greater than or equal to that produced by A-PPD. Using the specific antigens as a proteic cocktail, the specificity and sensitivity reached 100% considering the new relative and absolute cut-offs in all experimental groups (Δ≥30% and Δmm ≥ 2, respectively). Results suggest that the interference caused by paratuberculosis vaccination in cattle could be completely overcome by applying new approaches to the official tuberculosis diagnostic tests.

## Introduction

Bovine tuberculosis (bTB) and paratuberculosis (PTB) are widespread infectious diseases that affect many domestic [1,2] and wild [3,4] species. The impact of these diseases derives from losses to the livestock industry, especially dairy cattle [5,6], from hunting and wildlife conservation as well as from their recognized (bTB) or suspected (PTB) zoonotic character. The relevance of bTB as a zoonosis has been substantially reduced in the more developed countries, but it is still a frequent cause of morbidity and mortality in countries that cannot afford strong control measures like milk thermal treatment and compulsory bTB eradication schedules [6]. It has been estimated that about 10% of the total human tuberculosis cases around the world are caused by *Mycobacterium bovis (M*. *bovis)* [7,8]. On the other hand *Mycobacterium avium* subsp. *paratuberculosis* (*Map*) is considered potentially zoonotic since it was first isolated from human patients in 1984 [9] and has also been firmly associated with some forms of chronic regional intestinal inflammatory disease [10–12], although an etiological role has not been widely accepted by the medical community.

The live *M*. *bovis* Bacillus Calmette-Guerin (BCG) has been used as a vaccine in humans [6] as well as in cattle [13], showing different levels of protection against *M*. *bovis* infection. One of the major disadvantages of the use of this attenuated vaccine in cattle is the interference with bTB diagnostic tools due to cross reactivity of the tuberculins with antigens of the vaccine itself.

PTB is considered one of the most important diseases in dairy cattle, decreasing the milk production by up to 10% [5,14]. It has been proven that PTB vaccination in sheep and goats with *Map* whole heat-inactivated vaccines efficiently prevents the disease and significantly diminishes the bacterial burden reducing the chance for other animals of becoming infected [15]. As a consequence PTB vaccination should be taken into account in countries where bTB prevalence is really low and the Animal Health System works efficiently [16].

In Spain, PTB vaccination is not allowed in cattle due to the possible interference in the official immunological bTB diagnostic tests [17]. bTB herd prevalence in Spain is 1.2% [18] but the Basque Country is one of the regions with lowest frequencies (0.25%) [19] and it can be very closely monitored thanks to its small size and well developed veterinarian services. For these reasons a field clinical trial for an inactivated vaccine registration was authorized by the competent authorities (local Animal Health and Animal Experimentation Authority, the Spanish Drug Registration Authority and the Central Animal Health Authority) whose results have been partially published [16,17,20]

Moreover, simultaneous infection of herds with *Map* and *M*. *bovis* may occur [21] and it may be responsible for a reduced sensitivity (Se) of the cell-mediated immune (CMI) response-based tests to detect bTB [22]. Over the last few years, antigens that are present in *M*. *bovis* but absent in both: tuberculosis (BCG) and PTB vaccines, such as ESAT-6/CFP-10 or Rv3615c, have been assayed as an alternative to avian and bovine purified protein derivatives (A-PPD and B-PPD), traditionally used in the current Comparative Intradermal Test (CIT) and interferon (IFN)-gamma assays [23].

Another issue that may affect bTB diagnosis is exposure to environmental mycobacteria, especially *Mycobacterium avium avium (M*. *a*. *avium)*. Previous studies have concluded that exposure to *M*. *a*. *avium* may impart a degree of immunity to *M*. *bovis* infection that can compromise currently used diagnostic tests, making improvement of test Se dependent on the use of specific antigens [24]. Regarding vaccine protection, some authors have concluded that sensitization with environmental mycobacteria may adversely affect the efficacy of the BCG vaccination [25] whereas others suggest that there is no evidence that natural pre-exposure to *M*. *avium* reduces the effectiveness of BCG vaccination [26] and that it rather causes an overall protection that cannot be further increased by vaccination.

The goal of this work was to assess different strategies to avoid PTB vaccination interference with CMI response-based bTB detection tests in cattle experimentally challenged by the use of both: alternative interpretation criteria for the standard comparative intradermal test and new more specific antigens [24].

## Materials and Methods

### Calves, inclusion criteria and vaccination

**Fig 1:**
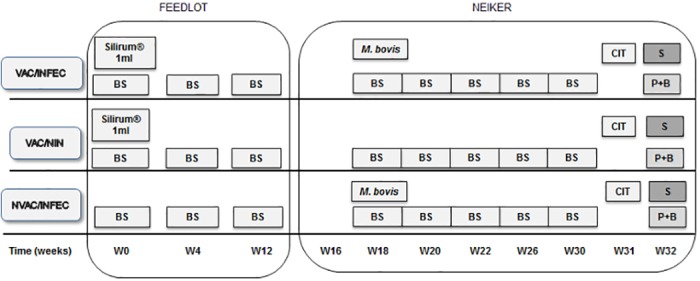
Experimental scheme. VAC/INFEC: vaccinated infected group. VAC/NIN: vaccinated non-infected group. NVAC/INFEC: non-vaccinated infected group BS: Blood Sampling. S: Slaughter. P+B: Pathology and Bacteriology. CIT: Comparative Intradermal Test. W: Week.

The experimental scheme is detailed on Fig 1. Thirty Fresian calves from a feedlot were preselected to carry out this experiment. These animals were born in 13 different farms located in Northern Spain. Two months after birth, at week 0 (W0), animals were submitted to a IFN-gamma release assay with standard (A-PPD, B-PPD) or more specific (ESAT-6/CFP10 and Rv3615c) antigens for the diagnosis of bTB. Immediately afterwards, 20 of them were subcutaneously vaccinated against *Map* with 1ml of a heat inactivated vaccine (Silirum^®^ CZV, Porriño, Pontevedra, Spain) in the dewlap [17]. To confirm absence of contact with *M*. *bovis*, the samplings were repeated twice (W4, W12). Only 15 animals: 10 vaccinated and 5 non-vaccinated were kept and transferred to the biosafety level 3 (BSL-3) facilities in NEIKER. After arrival, calves were split into three separate groups of five animals each. The 15 remaining calves were kept in the feedlot and not further followed.

### *M*. *bovis* challenge

All the experimental procedures were carried out according to the European, National and Regional Law and Ethics Committee regulations. The experimental design underwent ethical review and approval by NEIKER’s Animal Care and Use Committee and by the Agriculture Department (PARAPATO-1264-BFA). Once animals were transferred to the BSL-3 facilities, they went through a two-week adaptation period. At W18 animals were sampled and five vaccinated and five non-vaccinated animals were challenged with 10^6^ colony forming units (CFU) of an *M*. *bovis* field isolate suspended in 2 ml of phosphate-buffered saline (PBS) by the endotracheal route. The isolate used for challenge was originally obtained from a naturally infected cow and identified as *M*. *bovis* spoligotype profile SB0339 according to the *M*.*bovis* Spoligotype Database website (www.mbovis.org). Prior to challenge, animals underwent intramuscular sedation with Xylazine (10 mg/50 kg). The final experimental groups were: PTB vaccinated and *M*. *bovis* infected (VAC/INFEC), PTB vaccinated and *M*. *bovis* non-infected (VAC/NIN), and PTB non vaccinated and *M*. *bovis* infected (NVAC/INFEC).

### Blood sampling

Blood was collected from the jugular or caudal vein at W0, W4, W12, W18, W20, W22, W26 and W30 in tubes with lithium heparin and immediately aliquoted and processed in the laboratory.

### Diagnostic tests

#### IFN-gamma release assay

Stimulation of whole blood with B-PPD, A-PPD and defined antigens, ESAT-6/CFP-10 and Rv3615, was carried out within 8 hours after sample collection. Five 1.5 ml aliquots of whole blood from each animal were stimulated with: 100 μl of PBS, 100 μl of A- and B-PPD(20μg/ml as assay concentration) (CZV, Porriño, Pontevedra, Spain) respectively, and with 150 μl (to achieve an assay concentration of 5 μg/ml each peptide) of ESAT-6/CFP10 and Rv3615c antigens that were tested as sets of overlapping peptides representing either ESAT-6/CFP-10 in one peptide cocktail or, alternatively, a peptide cocktail representing Rv3615c [27]. After incubating the plates for 16-24h at 37°C 5–7%CO_2_ the stimulated samples were centrifuged and subsequently the plasma was separated.

Quantification of IFN-gamma concentration in the plasma’s supernatant was performed by ELISA using the Bovigam^®^ commercial kit according to the manufacturer’s instructions (Prionics, Schlieren, Switzerland).

For result analysis, our first approach was to represent mean optical density (OD) values for each antigen to compare the treatment groups. The OD of the non-stimulated samples was subtracted from the OD of those stimulated with the different antigens. Afterwards, OD cut-offs were established for each defined *M*. *bovis* antigen, and frequencies of positive results were calculated and compared among groups.

Cut-offs alternative to the currently established ones for the standard antigens were studied for this experiment. Also, considering that our group of interest was the VAC/NIN, a new cut-off for the specific antigen (ESAT-6/CFP10) different from the previously defined cut-off by Vordermeier *et al*. [28], was selected. The goal was to find cut-offs that would allow us to improve and maximize both: Se and Sp of the diagnostic techniques. This approach which will be thoroughly explained in the results section.

#### Comparative intradermal test (CIT)

The CIT was carried out at W3, this is, one week before slaughter. The test was performed according to the European Communities Commission Regulations (regulation1226/2002, amending annexes A and B of the consolidated Council Directive 64/432/EEC) and the Royal Decree RD2611/1996 by the Official Veterinary Services inoculating 0.1 ml of B-PPD and A-PPD. In addition to the standard PPD antigens, 0.1 ml of a peptide cocktail (100 μg/ml/peptide) and a protein cocktail (100 μg/ml/protein) representing ESAT-6/CFP10 and Rv3615c were also used. Four sites on the necks of the animals were selected and the skin thickness of every injection site was measured before and 72 h after the inoculation. The interpretation was carried out according to official criteria (EU Council Directive 64/432/CEE and RD 2611/1996).

Since standard criteria showed lower Se or Sp in vaccinated animals, alternative criteria were studied for skin test interpretation in the different tests regarding not only the absolute but also the relative skin thickness increase threshold and antigen comparison. The obtained outcomes are shown in the result section.

#### Post-mortem studies

The animals were slaughtered at W32 in three consecutive days, five calves per day. The animals underwent sedation with XILAGESIC^®^ 2% (10 mg/ 50 kg l.w) (Laboratorios Calier, S.A., Barcelona, Spain) and then euthanized by an intravenous injection of T61 (4-6ml/50kg). Complete necropsies were carried out and samples were collected from several organs (lymph nodes, lung, tonsils, liver and kidney) for histopathological and microbiological analysis.

#### Data analysis

Frequency of positive results in each group/technique/antigen and time was used as a qualitative variable for diagnostic time dynamics description and group Se and Sp estimates comparison.

## Results

### Success of challenge procedure

Infection was achieved in all challenged animals as all were confirmed infected by bacteriological and pathological analysis. VAC/NIN animals did not present lesions compatible with bTB, whereas all the animals belonging to the challenged groups did. All isolates displayed the same *M*. *bovis* spoligotype as the challenge strain. Post-mortem findings will be reported elsewhere.

#### IFN-gamma

The mean value for every IFN-gamma result obtained was calculated. If the outcome for the B-PPD was ≥ 0.1 and B-PPD>A-PPD or Rv3615c was ≥ 0.1 or ≥ 0.3 for ESAT-6/CFP10 the results were considered *M*. *bovis* positive, respectively.

**Fig 2:**
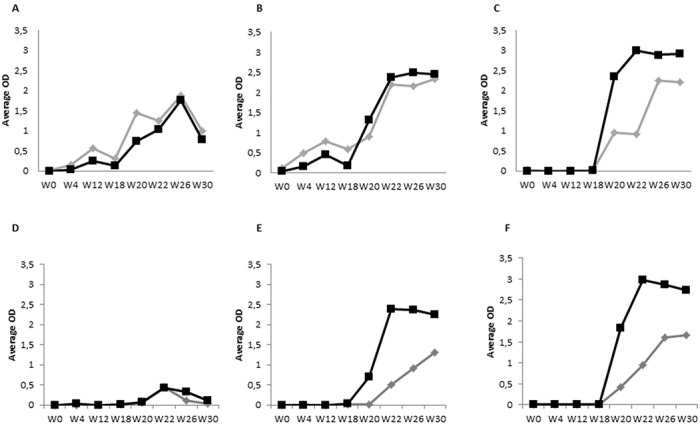
Cellular immune response measured as IFN-gamma release. Avian-PPD (gray rhombus), Bovine-PPD (black square) (A, B, C), ESAT-6/CFP10 (black square) and Rv3615c (gray rhombus) (D, E, F). Groups: vaccinated non-infected (VAC/NIN) (A and D), vaccinated infected (VAC/INFEC) (B and E) and non-vaccinated infected (NVAC/INFEC) (C and F).

Evolution of average IFN-gamma release over time per group and antigen is shown on Fig 2. The IFN-gamma levels upon stimulation with standard antigens A-PPD and B-PPD (Fig 2A, 2B and 2C) showed slight response increase in both vaccinated groups (VAC/NIN and VAC/INFEC) at W12, whereas the NVAC/INFEC group did not respond to standard antigens during the pre-infection period. However, upon challenge, reactions against A-PPD and B-PPD rose in all three groups of animals. The VAC/NIN (Fig 2A) group showed a heterogeneous response with peaks and troughs from W18 to W26, always higher to the A-PPD than to the B-PPD and declining sharply in both cases after W26. On the contrary, the NVAC/INFEC group (Fig 2C) showed an increasing response following B-PPD stimulation throughout the post-infection period.

The response to the defined antigens showed higher definition than the standard ones as seen in Fig 2D, 2E and 2F. During the pre-challenge period none of the animals in any of the groups showed any kind of response against ESAT-6/CFP10 or Rv3615c. However, infection immediately triggered a response in both infected groups (VAC/INFEC and NVAC/INFEC) (Fig 2E and 2F) although the NVAC/INFEC animals (Fig 2F) showed slightly higher IFN-gamma levels compared to the VAC/INFEC animals (Fig 2E). Surprisingly, the VAC/NIN group (Fig 2D) showed a minimum response at W22. ESAT-6/CFP10 showed higher discrimination power since differences between infected and non-infected groups were greater.

The qualitative results for the IFN-gamma release test are shown on Table 1. The B-PPD detected all infected animals in the NVAC/INFEC during the post-infection period, showing different degrees of detection in the VAC/INFEC group and slight cross reactivity in some of the VAC/NIN animals. Applying the calculated cut-offs for the defined *M*. *bovis* antigens, in the VAC/NIN group a slight non-specific reaction could be observed for both during the post-infection weeks although infection was discarded according to histopathological and microbiological results. This behavior tended to disappear in the last sampling at W30. These defined specific antigens exhibited a mild delay in the detection of infected animals in the VAC/INFEC group. Regarding Rv3615c, the reactivity seemed to decrease at W30. Both, Rv3615c and ESAT-6/CFP10 peptide cocktails were equally efficient at classifying animals from the NVAC/INFEC group from W22 on.

**Table 1:** Percentage of positive animals for interferon-γ testing.

State	Antigen	W0	W4	W12	W18	W20	W22	W26	W30
**VAC/NIN**	B-A	0%	0%	0%	0%	0%	20%	40%	20%
	Rv3615c	0%	0%	0%	0%	20%	100%	20%	0%
	ESAT-6/CFP10	0%	0%	0%	0%	0%	60%	20%	0%
**VAC/INFEC**	B-A	0%	0%	0%	0%	100%	40%	40%	80%
	Rv3615c	0%	0%	0%	0%	0%	80%	100%	80%
	ESAT-6/CFP10	0%	0%	0%	0%	80%	100%	100%	100%
**NVAC/INFEC**	B-A	0%	0%	0%	0%	100%	100%	100%	100%
	Rv3615c	0%	0%	0%	0%	80%	100%	100%	100%
	ESAT-6/CFP10	0%	0%	0%	0%	100%	100%	100%	100%

VAC/NIN: vaccinated non-infected group. VAC/INFEC: vaccinated infected group. NVAC/INFEC: non-vaccinated infected group. W: week. The Optical Density (OD) cut-off for Rv3615 –PBS is >0.1 and > 0.3 for ESAT-6/CFP10—PBS. For the standard antigens the meet requirements are B-PPD > A-PPD and OD for B-PPD—PBS is > 0.1. Vaccination was performed at W0 and experimental challenge at W18.

#### Comparative Intradermal Test (CIT)

Skin test raw data and interpretation are detailed on Table 2. All calves included in the VAC/NIN group (5/5) showed a PPD-A response bias and were therefore classified as CIT-negative. However, substantial responses were recorded to PPD-B which meant that 3/5 calves were classified as SIT-positive (Table 2).

**Table 2:** Intradermal Response: raw data and interpretation.

				Interpretation		ESAT-6/CFP10/Rv3615c cocktail (Δmm interpretation)			
State	Calf	Avian PPD (Δmm)	Bovine PPD (Δmm)	SIT	CIT	Peptide		Protein	
**VAC/NIN**	**1**	12	5	+	N	0	N	0	N
	**2**	14	5	+	N	3	+	0	N
	**3**	9	3	N	N	1	N	0	N
	**4**	6	4	+	N	0	N	0	N
	**5**	8	3	N	N	0	N	0	N
**VAC/INFEC**	**6**	9	9	+	N	3	+	5	+
	**7**	7	6	+	N	6	+	4	+
	**8**	12	23	+	+	11	+	15	+
	**9**	11	10	+	N	6	+	3	+
	**10**	8	18	+	+	7	+	7	+
**NVAC/INFEC**	**11**	2	9	+	+	5	+	6	+
	**12**	1	8	+	+	8	+	6	+
	**13**	1	12	+	+	9	+	7	+
	**14**	0	9	+	+	3	+	3	+
	**15**	1	5	+	N	4	+	6	+

VAC/NIN: vaccinated non infected group. NVAC/INFEC: non vaccinated infected group. VAC/INFEC: vaccinated infected group. PPD: protein purified derivative. SIT: single intradermal test, CIT: comparative intradermal test. N: negative. +: positive. Δmm: skin thickness increase in mm. The SIT is considered positive when an increase bigger than 4mm is observed in the PPD-B injection site after 72h The CIT is considered positive when the skin thickness increase at the PPD-B injection site is 4 mm greater than the increase induced at the PPD-A injection site after 72h. A ESAT-6/CFP10/Rv3615c peptide or protein cocktail result will be considered positive if the skin thickness increase is equal to or bigger than 2mm.

The skin test was performed using two more antigenic preparations: ESAT-6/CFP10-Rv3615c as a peptide or protein cocktail. We considered a skin increase of 2 mm or greater as a positive result. Using this cut-off value, 1/5 VAC/NIN animals tested positive to the ESAT-6/CFP10-Rv3615c peptide cocktail while none responded to the protein cocktail (0/5, Table 2). Taken together, while the Sp of the SIT is compromised by PTB vaccination, this limitation can be overcome by using the CIT or defined protein antigens as skin test reagent.

Following *M*. *bovis* infection, the detection (Se) of infection in the PTB vaccinated group (VAC/INFEC) was severely compromised as 3/5 animals escaped CIT detection due to the PPD-A biased responses (Table 2) as well as in the non-vaccinated/infected group (NVAC/INFEC) were an animal out of 5 was considered as a negative one (4/5 Table 2). As discussed, the SIT is severely compromised in respect of specificity following PTB vaccination, and therefore the observation that it detected all infected animals regardless of their vaccination status is irrelevant (Table 2, VAC/INFEC and NVAC/INFEC groups, 10/10, Table 2). In marked contrast, the ability of the defined antigen reagents to detect *M*. *bovis* infected calves was not affected by their vaccination status, as 10/10 animals in the VAC/INFEC and NVAC/INF groups were detected by protein and peptide cocktails (Table 2).

Table 3 shows the relative increase of the skin thickness 72h after the intradermal inoculation. The VAC/INFEC group showed the greatest (Mean = 230%) relative skin increase to the B-PPD followed by the NVAC/INFEC while the VAC/NIN group exhibited the smallest (Mean = 63.8%) skin relative increase with the B-PPD. The A-PPD showed the greatest skin increase in the vaccinated groups (VAC/INFEC (Mean = 127.4%) and VAC/NIN (Mean = 128.6%)). Both, peptide and protein cocktails showed highest skin increase percentages in the infected groups, (VAC/INFEC: Mean = 87.6% and Mean = 101%; NVAC/INFEC: Mean = 92.6%; and Mean = 86.4% respectively) (Table 3).

**Table 3:** Individual skin test results: skin thickness increase relative to the initial reading.

				ESAT-6/CFP10/Rv3615c	
		Avian PPD	Bovine PPD	Peptide cocktail	Protein cocktail
State	Calf	Δ%	Δ%	Δ%	Δ%
**VAC/NIN**	**1**	120	63	0	0
	**2**	140	56	30	0
	**3**	150	60	17	0
	**4**	100	80	0	0
	**5**	133	60	0	0
	**MV =**	128,6	63,8	9,4	0
**VAC/INFEC**	**6**	150	150	43	71
	**7**	140	150	120	100
	**8**	109	383	100	167
	**9**	138	167	75	50
	**10**	100	300	100	117
	**MV =**	127,4	230	87,6	101
**NVAC/INFEC**	**11**	33	180	83	120
	**12**	13	133	133	100
	**13**	13	171	180	88
	**14**	0	113	27	38
	**15**	17	62	40	86
	**MV =**	15,2	131,8	92,6	86,4

VAC/NIN: vaccinated non infected group. NVAC/INFEC: non vaccinated infected group. VAC/INFEC: vaccinated infected group. Δ%: skin thickness increase at 72 h after inoculation expressed as a percentage. MV: mean value

Table 4 shows the performance of the different alternative diagnostic criteria. A SIT interpretation with an absolute skin thickness increase of 6 mm or more detected all the VAC/INFEC animals but none of the VAC/NIN, accounting for 100% Se and Sp.

**Table 4:** Skin thickness interpretation criteria after application of the different antigens in the skin test.

Antigen	Test type and interpretation	Cut-off positivity criteria	Sensitivity %	Specificity %
**Official diagnostic criteria: vaccinated and non-vaccinated animals**				
**PPD**	Single official	Standard: Δmm B-PPD≥4	100	40
		Strict: Δmm B-PPD>2	100	0
	Comparative official	Standard: Δmm B-PPD≥4 and >A-PPD	70	100
		Strict: Δmm B-PPD>2 and >A-PPD	70	100
**Alternative diagnostic criteria (vaccinated animals only)**				
**PPD**	Single absolute	Δmm ≥ 6	100	100
	Comparative absolute	Δmm ≥ 6 and B-PPD ≥ A-PPD	60	100
	Single relative	≥ 100%	100	100
	Comparative relative	≥ 100% and B-PPD ≥ A-PPD	100	100
**Peptide cocktail**	Single absolute	Δmm ≥ 3	100	80
	Single relative	≥ 40%	100	100
**Protein cocktail**	Single absolute	Δmm ≥ 3	100	100
	Single relative	≥ 30%	100	100
**Alternative diagnostic criteria (non-vaccinated animals only)**				
**Peptide cocktail**	Single absolute	Δmm ≥ 3	100	100
**Protein cocktail**	Single absolute	Δmm ≥ 3	100	100

A-PPD: avian purified protein derivative. B-PPD: bovine purified protein derivative.

With a comparative interpretation where an increase of 6 mm in the B-PPD that was equal to or greater than the A-PPD, all VAC/NIN were correctly classified as negative, but only 3/5 of the VAC/INFEC were scored as positive. Thus the Se was 60% and the Sp 100%.

In order to establish criteria, focus was set on the vaccinated groups because the aim of this work was to set up the parameters to be able to differentiate VAC/INFEC animals from those which were only PTB-vaccinated. Regarding relative readings, for the SIT, a cut-off at a 100% skin increase classified all the animals in the VAC/INFEC as bTB positive, while all in the VAC/NIN group were classified as non-bTB reactors (Table 3). This yielded 100% Se and 100% Sp (Table 4). A comparative reading, scoring as positive a B-PPD relative increase greater than the A-PPD increase, also yielded 100% Se and Sp. Using the protein cocktail two different cut-offs were established. The first one takes into account the absolute skin increase and the second the relative. Despite the low number of animals belonging to each group, these results are very interesting since all the animals were correctly classified.

## Discussion

The main aim of this study was to assess the interference of an inactivated PTB vaccine with the official bTB diagnostic tests. In order to improve the diagnostic efficacy of the CMI-based test, the immunological response of the animals was determined comparing the standard antigens PPD-A and PPD-B with two different formulations of defined antigens (ESAT-6/CFP10 and Rv3615c) [29]. In addition, new criteria for result interpretation according to the vaccination status of animals were explored.

During the last decade great efforts have been dedicated to the study and development of new specific antigens [29,30] that have been thoroughly assessed by *in vitro* IFN-gamma tests [31]. The use of these antigens has received attention for the skin test [32] in PTB vaccinated animals, and it has been proven that protein [23,32] and peptidic cocktails [31] can be suitable under field conditions.

Furthermore, to the best of our knowledge, this is the first time that the skin test has been performed with these specific cocktails in PTB vaccinated animals and subsequently *M*. *bovis* infected cattle, although these antigens had been previously shown not to be recognized following PTB vaccination in goats [33]. (Jones et al., CVI 2012).

The *M*. *bovis* challenge process was carried out successfully; all the calves belonging to infected groups became infected regardless of their vaccination status. However, the severity and extension of the lesions was lower in the VAC/INFEC animals (data not shown). These results are in agreement with the findings from Pérez del Val *et al* [33] who also reported that *Map* vaccinated and *M*. *bovis* infected goats showed minor severity and extension of the lesions in an experimental setting, suggesting that PTB vaccination could provide a certain degree of containment of bTB dissemination [33].

The dynamics of the IFN-gamma test results shows that PTB vaccination induced a response against standard PPD mycobacterial antigens that was predominantly of the avian type before to *M*. *bovis* challenge. Although a noticeable degree of cross reactivity to B-PPD was observed it did not affect the Sp of the CIT. After infection, all groups switched to a predominantly bovine PPD-biased response that was slightly lower in the vaccinated group compared to the non-vaccinated group. All the animals belonging to the VAC/INFEC group were categorized as infected only in the first sampling post-challenge at W20, while all NVAC/INFEC animals were positive throughout the complete post-infection period. This indicates that IFN-gamma test did not work well in vaccinated animals in terms of Se and also Sp.

The more specific antigens worked better in terms of Se, in particular ESAT-6/CFP10 that only failed to detect one animal on W20, two weeks post challenge. It was also the most specific since it only yielded false positive results in the non-infected group for two animals four weeks post-infection (W22) and for one calf eight weeks post-infection (W26). The movement of the animals to the P3 facilities might have changed the microbial environment and induced an unspecific cellular immune response that reached a certain development in the VAC/NIN group, but that was quickly replaced by a more specific one in the animals that were challenged with *M*. *bovis*.

Results have demonstrated that the response pattern of the traditional antigens versus the more specific ones can be very different. The ESAT-6/CFP10 and Rv3615 antigens did not show any kind of response during the post-vaccination period and they just underwent a raise in their response after *M*. *bovis* challenge. A-PPD and B-PPD responses based on the IFN-gamma release made differentiation difficult especially among the VAC/INFEC animals. Similar results have been observed with the same antigens in goats [33].

In this experiment we decided to apply 2 interpretation criteria for the qualitative IFN-gamma results: one for the B-PPD and a second one for the specific antigens responses. The most extended OD cut-off point (0.1) was selected for the B-PPD. For the ESAT-6/CFP10 the OD cut-off was set at 0.3 and at 0.1 for Rv3615c. Selecting these cut-offs, the Se of the technique in the most problematic group (VAC/INFEC) reached 100% when the ESAT-6/CFP10 antigen was used. This Se rate was maintained from the second post-infection sampling throughout the experiment. Our data showed that the Se obtained with this specific antigen was higher than the one obtained with B-PPD as previous researchers demonstrated [23,34].

Regarding the conventional CIT results, all the animals belonging to the VAC/NIN and NVAC/INFEC groups were correctly classified in relation to the infectious status according to treatment and necropsy results. The interference problem arose when the VAC/INFEC group has to be diagnosed using the same criteria. Applying the traditional comparative skin test 60% of the VAC/INFEC animals were misclassified as *M*. *bovis* non-infected.

If new cut-off points were set up, this drawback could be overcome. Regarding the vaccinated and non-vaccinated groups alternative cut-offs were established for every antigen studied (Table 4). As it can be observed, from all the assessed possibilities, those which measured the relative increase of the skin thickness were the most reliable in both vaccinated groups, in terms of Se and Sp.

When the non-vaccinated animals were taken into account, whether the chosen diagnostic interpretation is the official criterion or the new criterion, 100% of the animals were diagnosed correctly in relation to our gold standard: the necropsy results. These outcomes reinforce the idea that our efforts should focus on validating a technique able to identify all the animals from the problematic group, VAC/INFEC animals.

In spite of the low number of animals and that they were kept under experimental conditions, the Se and Sp obtained (100% respectively) when using the protein cocktail in the skin test were very encouraging. All the animals belonging to the three different groups were correctly classified. That is why in view of the outstanding results it would be of great interest to test the protein cocktail under field conditions.

In this experimental scheme, we have demonstrated that it is possible to differentiate *M*. *bovis* infected animals even after PTB vaccination by including the specific antigens into the skin test, or by adopting new interpretation criteria for the conventional ones. These findings lend support to the PTB vaccination strategies showing that vaccination in bTB affected environments should not be a problem.

## Conclusions

Our results prove that the ESAT-6/CFP10 and Rv3615c proteinic and peptidic cocktails can be used as skin test reagents in the face of PTB vaccination and *M*. *bovis* infection without compromising either Se or Sp. In respect to IFN-gamma results, use of these defined antigens improved the Se and Sp compared to the conventional antigens A-PPD and B-PPD. In conclusion PTB vaccination produces interference in cattle experimentally infected with *M*. *bovis*, but this could be overcome if new testing strategies such as new specific antigens were applied alternative to or complementing the official bTB diagnostic tests or if new diagnostic criteria with traditional antigens were established. Although the results presented are promising, this study has been performed in experimental bTB infection conditions and they should be further validated in field conditions.
